# Distinctive electrophysiological characteristics of right ventricular out-flow tract cardiomyocytes

**DOI:** 10.1111/jcmm.12329

**Published:** 2014-06-09

**Authors:** Yen-Yu Lu, Fa-Po Chung, Yao-Chang Chen, Chin-Feng Tsai, Yu-Hsun Kao, Tze-Fan Chao, Jen-Hung Huang, Shih-Ann Chen, Yi-Jen Chen

**Affiliations:** a Graduate Institute of Clinical Medicine, College of Medicine, Taipei Medical UniversityTaipei, Taiwan; b Division of Cardiology, Department of Internal Medicine, Sijhih Cathay General HospitalNew Taipei City, Taiwan; c Division of Cardiology and Cardiovascular Research Center, Veterans General Hospital-TaipeiTaipei, Taiwan; d Department of Biomedical Engineering, National Defense Medical CenterTaipei, Taiwan; e Division of Cardiology, Department of Internal Medicine, Chung Shan Medical University Hospital, Chung Shan Medical UniversityTaichung, Taiwan; f Department of Medical Education and Research, Wan Fang Hospital, Taipei Medical UniversityTaipei, Taiwan; g Division of Cardiovascular Medicine, Department of Internal Medicine, Wan Fang Hospital, Taipei Medical UniversityTaipei, Taiwan; h School of Medicine, National Yang-Ming UniversityTaipei, Taiwan

**Keywords:** arrhythmogenicity, calcium handling, right ventricular out-flow tract, ventricular arrhythmias

## Abstract

Ventricular arrhythmias commonly originate from the right ventricular out-flow tract (RVOT). However, the electrophysiological characteristics and Ca^2+^ homoeostasis of RVOT cardiomyocytes remain unclear. Whole-cell patch clamp and indo-1 fluorometric ratio techniques were used to investigate action potentials, Ca^2+^ homoeostasis and ionic currents in isolated cardiomyocytes from the rabbit RVOT and right ventricular apex (RVA). Conventional microelectrodes were used to record the electrical activity before and after (KN-93, a Ca^2+^/calmodulin-dependent kinase II inhibitor, or ranolazine, a late sodium current inhibitor) treatment in RVOT and RVA tissue preparations under electrical pacing and ouabain (Na^+^/K^+^ ATPase inhibitor) administration. In contrast to RVA cardiomyocytes, RVOT cardiomyocytes were characterized by longer action potential duration measured at 90% and 50% repolarization, larger Ca^2+^ transients, higher Ca^2+^ stores, higher late Na^+^ and transient outward K^+^ currents, but smaller delayed rectifier K^+^, L-type Ca^2+^ currents and Na^+^-Ca^2+^ exchanger currents. RVOT cardiomyocytes showed significantly more pacing-induced delayed afterdepolarizations (22% *versus* 0%, *P* < 0.05) and ouabain-induced ventricular arrhythmias (94% *versus* 61%, *P* < 0.05) than RVA cardiomyocytes. Consistently, it took longer time (9 ± 1 *versus* 4 ± 1 min., *P* < 0.05) to eliminate ouabain-induced ventricular arrhythmias after application of KN-93 (but not ranolazine) in the RVOT in comparison with the RVA. These results indicate that RVOT cardiomyocytes have distinct electrophysiological characteristics with longer AP duration and greater Ca^2+^ content, which could contribute to the high RVOT arrhythmogenic activity.

## Introduction

Right ventricular out-flow tract (RVOT) is an important focus, from which ventricular tachyarrhythmias, such as idiopathic ventricular tachycardia (VT), ventricular arrhythmia with Brugada syndrome and torsade de pointes, commonly originate [1–3]. Functional dynamics of action potentials (APs) and repolarization heterogeneity in the RVOT are believed to be major contributors to Brugada-type electrocardiographic changes [4,5]. For instance, idiopathic VT can be caused by triggered activity from the RVOT as a consequence of increased intracellular Ca^2+^ and/or cAMP [6,7]. Other factors such as electrophysiological heterogeneities existing between the RVOT and right ventricular free wall [8], including regional differences in expression of connexins and ion channels [9], as well as distinct Ca^2+^ handling [10,11], have been proposed to play an important role in the RVOT arrhythmogenicity. However, the electrophysiological characteristics and Ca^2+^ regulation in RVOT cardiomyocytes have not been studied comprehensively.

The potential Ca^2+^ dysregulation in RVOT cardiomyocytes suggests that the RVOT may have a propensity for ouabain (a cardiac glycoside)-induced ventricular arrhythmias. Moreover, ouabain can also increase late sodium current (I_Na-Late_) through activation of Ca^2+^ calmodulin-dependent protein kinase II (CaMKII) [12], both of which play a key role in arrhythmogenesis by inducing early and delayed afterdepolarization (EAD/DAD) [13]. However, contributions of I_Na-Late_ and CaMKII to RVOT arrhythmogenesis are not fully elucidated. Ranolazine, an I_Na-Late_ inhibitor, which can suppress EADs and reduce transmural dispersion of repolarization [14], and decrease the VT burden [15]; and KN-93, a CaMKII inhibitor, which can prevent afterdepolarization, normalize L-type Ca^2+^ channel and suppress ventricular arrhythmias [16]. Therefore, the purpose of this study was to investigate the electrophysiological characteristics and Ca^2+^ homoeostasis in the RVOT in comparison with the RVA. We examined the propensity of RVOT cardiomyocytes to display dysrhythmic activity in response to cardiac glycoside and evaluate whether KN-93 or ranolazine can modulate RVOT arrhythmogenesis.

## Materials and methods

### Electrophysiological study of isolated single cardiomyocytes

**Fig. 1:**
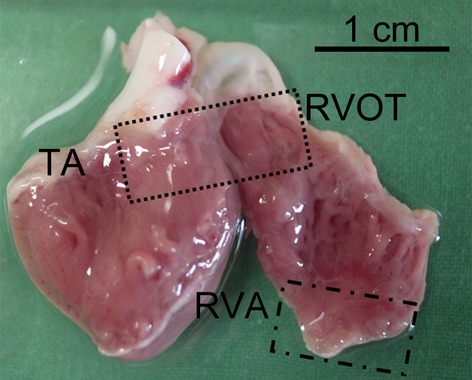
Experimental localizations of the right ventricular out-flow tract (RVOT) and right ventricular apex (RVA). TA indicates tricuspid annulus.

All animal experimental procedures were approved by the Institutional Animal Care and Use Committee (IACUC) at Taipei Veterans General Hospital (Protocol Number: IACUC 2011-028) and conformed to the institutional Guide for the Care and Use of Laboratory Animals and the Guide for the Care and Use of Laboratory Animals published by the United States National Institutes of Health (NIH Publication No. 85-23, revised 1996). Single RVOT and right ventricular apex (RVA) cardiomyocytes were isolated from 25 rabbits (weight 1–2 kg) anaesthetized with an intraperitoneal injection of sodium pentobarbital (100 mg/kg). Hearts were removed and mounted on a Langendorff apparatus to be superfused in an antegrade manner with oxygenated normal Tyrode's solution at 37°C, containing (in mM) NaCl 137, KCl 5.4, CaCl_2_ 1.8, MgCl_2_ 0.5, HEPES 10, and glucose 11; adjusted to pH 7.4 with NaOH. After the hearts were cleaned of blood, the perfusate was replaced with an oxygenated Ca^2+^-free Tyrode's solution containing 300 units/ml of collagenase type I (Sigma-Aldrich, St. Louis, MO, USA) and 0.25 units/ml protease type XIV (Sigma-Aldrich) for 8–12 min. The RVOT (area within 5 mm below the pulmonary valve) and RVA (area within 5 mm of the RVA) were excised (Fig. 1) and gently shaken in 50 ml of Ca^2+^-free oxygenated Tyrode's solution until single cardiomyocytes were obtained. The solution was then gradually changed to normal oxygenated Tyrode's solution. Cardiomyocytes were allowed to stabilize in the bath for at least 30 min. before the experiments.

### Measurement of intracellular Ca^2+^

Intracellular Ca^2+^ concentration ([Ca^2+^]_i_) was recorded by a fluorometric ratio technique as previously described [17]. The fluorescent indicator, indo-1, was loaded by incubating cardiomyocytes at room temperature for 20–30 min. with indo-1/AM (10 μmol/l; Sigma-Aldrich). Then, cardiomyocytes were then perfused with normal Tyrode's solution at 35 ± 1°C for ≥30 min. to wash out the extracellular indicator and allow for intracellular de-esterification of indo-1. Background and cellular autofluorescence levels were cancelled out by zeroing the output of the photomultiplier tubes by using cells without indo-1 loading. Ultraviolet light of 360 nm from a monochromator was used to excite indo-1 with a xenon arc lamp controlled by a microfluorometric system (OSP100-CA; Olympus, Tokyo, Japan). The excitation light beam was directed into an inverted microscope (IX-70; Olympus). The emitted fluorescence signals from indo-1/AM-loaded cardiomyocytes were digitized at 200 Hz. We calculated the ratio of fluorescence emissions at 410 and 485 nm. The R_410/485_ value was used as an index of [Ca^2+^]_i_ and measured during 1 Hz field stimulation. After achieving a steady-state Ca^2+^ transients with the repeated pulses from −40 to 0 mV (1 Hz for 5 sec.), the sarcoplasmic reticulum (SR) Ca^2+^ content was estimated by integrating the Na^+^-Ca^2+^ exchanger (NCX) current following application of 20 mM of caffeine within 0.5 sec. during rest with the membrane potential clamped to −40 mV to cause SR Ca^2+^ release [18]. The time integral of NCX current was converted to amoles (10^−18^ moles) of Ca^2+^ released from the SR [19].

### Ionic current measurements

Whole-cell patch clamp was used for single isolated cardiomyocytes with an Axopatch 1D amplifier (Axon Instruments, Foster City, CA, USA) at 35 ± 1°C [20]. The borosilicate glass electrodes (o.d., 1.8 mm) with tip resistances of 3–5 MΩ were used. Before the formation of the membrane-pipette seal, the tip potentials were zeroed in Tyrode's solution. Junction potentials between the bath and pipette solution (9 mV) were corrected for AP recordings. APs were recorded in the current-clamp mode, and ionic currents were measured in the voltage-clamp mode. A small hyperpolarizing step from a holding potential of −50 mV to a testing potential of −55 mV for 80 msec. was delivered at the beginning of each experiment. The area under the capacitative currents was divided by the applied voltage step to obtain whole-cell capacitance. Series resistance (R_s_) was compensated by 60–80%. APs were elicited from isolated cardiomyocytes without spontaneous activity at a driven rate of 1 Hz for 20 beats. The resting membrane potential (RMP) was measured during the period between the last repolarization and the onset of the subsequent AP. The AP amplitude (APA) was obtained from RMP to the peak of AP depolarization. AP duration at 90%, 50% and 20% repolarization were respectively measured as the APD_90_, APD_50_ and APD_20_. In cells with ‘spike and dome’ shape of APs, the magnitude of phase 1 notch was measured as the membrane potential between the peak of phase 0 and the end of phase 1 [21]. Micropipettes were filled with a solution containing (in mM) KCl 20, K aspartate 110, MgCl_2_ 1, MgATP 5, HEPES 10, EGTA 0.5, LiGTP 0.1 and Na_2_ phosphocreatine 5 (pH 7.2 with KOH).

I_Na_ was recorded by using 40 msec. pulses from a holding potential of −120 mV to the test potentials varying between −80 and 0 mV in 10 mV increments at a frequency of 3 Hz at room temperature (25 ± 1°C) The external solution contained (in mM): NaCl 5, CsCl 133, MgCl_2_ 2, CaCl_2_ 1.8, nifedipine 0.002, HEPES 5 and glucose 5 (pH 7.3). Micropipettes were filled with a solution containing (in mM) CsCl 133, NaCl 5, EGTA 10, MgATP 5, TEACl 20 and HEPES 5 (pH 7.3 with CsOH).

I_Na-Late_ was recorded at room temperature by using a step/ramp protocol consisting of a 100 msec. step to +20 mV (from a holding potential of −100 mV) followed by a 100 msec. ramp from +20 mV to −100 mV. I_Na-late_ was measured from the baseline to the peak of the tetrodotoxin (30 μM)-sensitive fraction of the current as described previously [22,23]. The external solution containing (in mM): NaCl 130, CsCl 5, MgCl_2_ 1, CaCl_2_ 1, HEPES 10 and glucose 10 at a pH of 7.4 with NaOH by a step/ramp protocol (−100 mV step to +20 mV for 100 msec., then ramp back to −100 mV over 100 msec.). Micropipettes were filled with a solution containing (in mM) CsCl 130, Na_2_ATP 4, MgCl_2_ 1, EGTA 10 and HEPES 5 (pH 7.3 with NaOH).

I_Ca-L_ was recorded by using 300 msec. pulses from a holding potential of −50 mV to test potentials varying between −40 and +60 mV in 10 mV increments at a frequency of 0.1 Hz. In the external solution, NaCl and KCl of the normal Tyrode's solution were replaced with TEACl and CsCl respectively. Micropipettes were filled with a solution containing (in mM) CsCl 130, MgCl_2_ 1, MgATP 5, HEPES 10, EGTA 10, NaGTP 0.1 and Na_2_ phosphocreatine 5 (pH 7.2 with CsOH). Steady-state inactivation of I_Ca-L_ was evaluated by using a standard protocol consisting of a 300 msec. pre-pulse and a 150 msec. test pulse. Peak current elicited by the test pulse was divided by the maximal current and plotted as a function of pre-pulse voltage. Data points were fitted with a Boltzmann function. Recovery from inactivation of I_Ca-L_ was assessed by using a two-pulse protocol with 200 msec. pre- and test pulses (from −80 to +10 mV) separated by varying time intervals. Data points were fitted with a single-exponential function.

NCX current was obtained as the nickel-sensitive current by subtracting current in the presence of 10 mM NiCl_2_ from that in control. The recording protocol consisted of 300 msec. pulses ranging from −100 to +100 mV from a holding potential of −40 mV at a frequency of 0.1 Hz. The external solution for the measurement of NCX contained (in mM) NaCl 140, CaCl_2_ 2, MgCl_2_ 1, HEPES 5 and glucose 10 (pH of 7.4). It was supplemented with strophanthidin (10 μM), nitrendipine (10 μM) and niflumic acid (100 μM). Micropipettes were filled with a solution containing (in mM) NaCl 20, CsCl 110, MgCl_2_ 0.4, CaCl_2_ 1.75, tetraethylammonium 20, 1,2-bis(2-aminophenoxy)ethane-*N*,*N*,*N*′,*N*′-tetraacetic acid (BAPTA) 5, glucose 5, MgATP 5 and HEPES 10 (pH of 7.25).

I_to_ was studied by using a protocol consisting of a 30 msec. pre-pulse from a holding potential of −80 to −40 mV to inactivate sodium channels followed by a 300 msec. test pulse to +60 mV in 10 mV increments at a frequency of 0.1 Hz in the presence of 200 μM CdCl_2_ in Ca^2+^-free normal Tyrode's solution as described previously [24]. Micropipettes were filled with the same as that used to record APs. I_to_ was measured as the difference between the peak outward current and the steady-state current [25]. Steady-state inactivation of I_to_ was evaluated by using a standard protocol consisting of a 1 sec. pre-pulse and a 0.15 sec. test pulse. Peak current elicited by the test pulse was divided by the maximal current and plotted as a function of pre-pulse voltage. Data points were fitted with a Boltzmann function. Recovery from inactivation of I_to_ was assessed by using a two-pulse protocol with 200 msec. pre- and test pulses (from −80 to +50 mV) separated by varying time intervals. Data points were fitted with a single-exponential function.

I_Kr-tail_ was measured as the outward peak tail current density following a 3 sec. pre-pulse from a holding potential of −40 mV to voltage between −40 and +60 mV in 10 mV steps at a frequency of 0.1 Hz in the presence of chromanol 293B (30 μM) and CdCl_2_ (200 μM) in the Ca^2+^-free normal Tyrode's solution. Micropipettes were filled with a solution containing (in mM) KCl 120, MgCl_2_ 5, CaCl_2_ 0.36, EGTA 5, HEPES 5, glucose 5, K_2_-ATP 5, Na_2_-CrP 5, Na-GTP 0.25 (pH 7.2 with KOH).

### Electropharmacological experiments in isolated RVOT and RVA tissue preparations

Tissue preparations (∼1 × 1.5 cm) of RVOT and RVA were isolated from 36 rabbits (weight 1–2 kg) anaesthetized with an intraperitoneal injection of sodium pentobarbital (100 mg/kg). Tissue preparations were bathed in Tyrode's solution at 37°C containing (in mM): NaCl 137, KCl 4, NaHCO_3_ 15, NaH_2_PO_4_ 0.5, MgCl_2_ 0.5, CaCl_2_ 2.7 and dextrose 11. The tissue was superfused at a constant rate (3 ml/min.) with Tyrode's solution, which was saturated with a 97% O_2_ and 3% CO_2_ gas mixture. Tissue preparations were connected to a WPI model FD223 electrometer by using a 150 mg load. The endocardial side of the preparations faced upwards, and the mechanical events were recorded by using Gould 4072 oscilloscope and Gould TA11 recorder (OH, USA). Signals were recorded digitally with a 16-bit accuracy at a rate of 125 kHz. For electrical stimulation, a 1 msec. pulse was provided by a Grass S88 stimulator through a Grass SIU5B stimulus isolation unit. The DAD was defined as a spontaneous depolarization event occurring after full repolarization.

Ouabain (0.1 and 1 μM) was used to superfuse the tissue for 30 min. Decremental electrical stimuli (1000, 500 and 200 msec.) were used to induce DADs and ventricular arrhythmias. After a sustained VT was induced by ouabain (1 μM), KN-93 (0.1 μM) or ranolazine (0.1 μM) was applied to the tissue at the constant rate. The duration of drug application required for suppressing the VT was measured.

### Statistical analysis

All quantitative results are shown as the mean ± SEM. Statistical significance between different groups was determined by using a paired or unpaired *t*-test, depending on the results of the normality test. Nominal variables were compared by a Chi-squared analysis with Pearson's correlation. A value of *P* < 0.05 was considered statistically significant.

## Results

### Electrophysiological characteristics and Ca^2+^ homoeostasis of RVOT and RVA cardiomyocytes

**Fig. 2:**
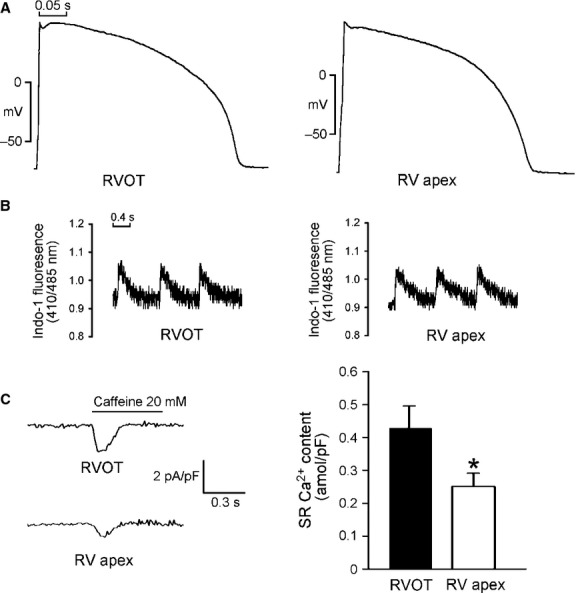
Action potential (AP) characteristics and Ca^2+^ homoeostasis of the right ventricular apex (RVA) and right ventricular out-flow tract (RVOT) cardiomyocytes. (**A**) Examples of the APs from RVA (*n* = 13) and RVOT (*n* = 16) cardiomyocytes. (**B**) Tracings from [Ca^2+^]_i_ transients in RVA (*n* = 23) and RVOT (*n* = 20) cardiomyocytes. (**C)** The tracings and average data of the caffeine-induced Na^+^-Ca^2+^ exchanger (NCX) currents and SR Ca^2+^ content from integrating the NCX currents in RVA (*n* = 17) and RVOT (*n* = 15) cardiomyocytes. *P < 0.05 *versus* RVOT.

As shown in Figure 2A, RVOT cardiomyocytes had longer APD_90_ (374 ± 16 msec. *versus* 325 ± 14 msec., *P* < 0.05) and APD_50_ (329 ± 15 msec. *versus* 286 ± 14 msec., *P* < 0.05) than RVA cardiomyocytes. RVOT and RVA cardiomyocytes had similar values for APD_20_ (175 ± 12 msec. *versus* 159 ± 18 msec., *P* > 0.05), APA (128 ± 1 mV *versus* 121 ± 4 mV, *P* > 0.05) and RMP (74 ± 1 mV *versus* 73 ± 2 mV, *P* > 0.05). Moreover, the phase 1 notch was more often observed in RVOT than RVA cardiomyocytes (58% *versus* 6%, *P* < 0.005), and its magnitude was larger in RVOT than in RVA cardiomyocytes (6.8 ± 2.1 mV *versus* 1.2 ± 1.2 mV, *P* < 0.05).

Figure 2B shows that [Ca^2+^]_i_ transients are on average by 24% larger in RVOT cardiomyocytes (0.15 ± 0.01 *versus* 0.12 ± 0.01, *P* < 0.05) than in RVA cardiomyocytes, and that RVOT cardiomyocytes are characterized by a relatively fast decay of [Ca^2+^]_i_ transients (130 ± 27 msec. *versus* 256 ± 49 msec., *P* < 0.05). The SR Ca^2+^ content was larger in RVOT cardiomyocytes than in RVA cardiomyocytes (Fig. 2C). These differences suggest that RVOT and RVA cardiomyocytes have different electrical activity and Ca^2+^ homoeostasis.

**Fig. 3:**
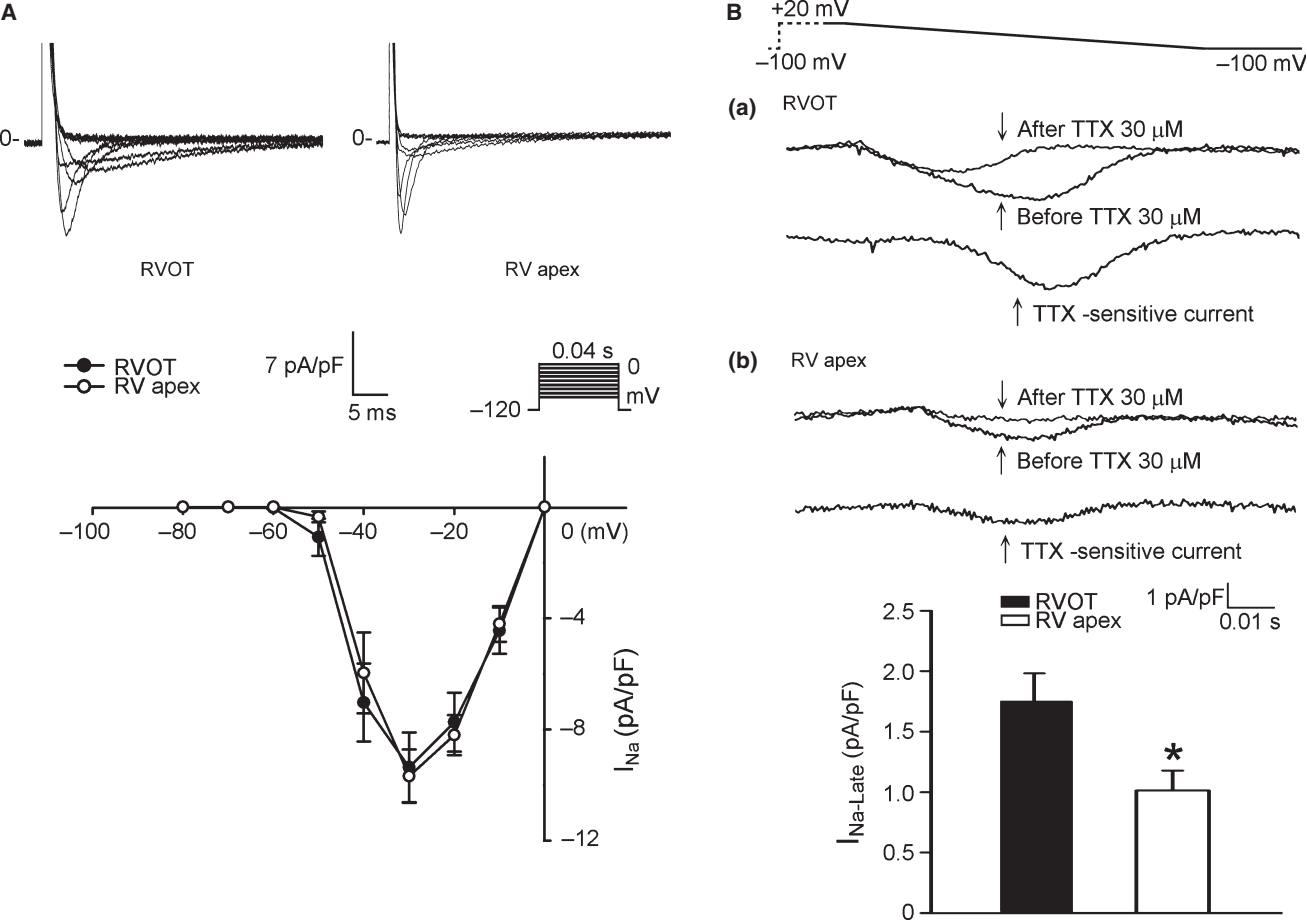
Sodium current (I_Na_) and late sodium current (I_Na-Late_) in right ventricular apex (RVA) and right ventricular out-flow tract (RVOT) cardiomyocytes. (**A**) Current tracing and I–V relationship of I_Na_ in RVA (*n* = 15) and RVOT (*n* = 15) cardiomyocytes. (**B**) The examples of current tracing and average data of I_Na-Late_ in RVOT (*n* = 17) and RVA (*n* = 13) cardiomyocytes. Insets in the current traces show the clamp protocol. *P < 0.05 *versus* RVOT.

**Fig. 4:**
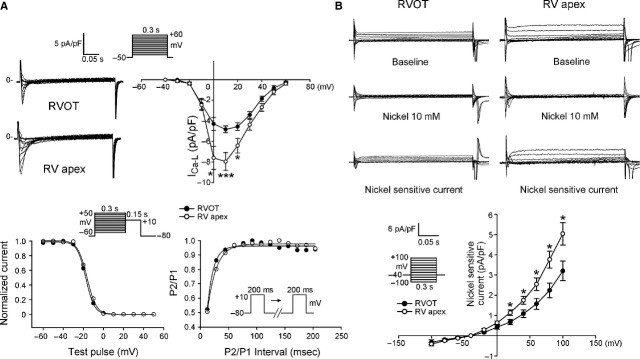
L-type Ca^2+^ current (I_Ca-L_) and nickel-sensitive Na^+^-Ca^2+^ exchanger (NCX) in right ventricular apex (RVA) and right ventricular out-flow tract (RVOT) cardiomyocytes. (**A**) Upper panels show the example of current traces and I–V relationship the I_Ca-L_ in RVOT (*n* = 18) and RVA (*n* = 18) cardiomyocytes. Lower panels show the voltage dependence of inactivation of I_Ca-L_ from RVOT (*n* = 11) and RVA (*n* = 14) cardiomyocytes and the recovery kinetics of I_Ca-L_ from RVOT (*n* = 12) and RVA (*n* = 17) cardiomyocytes. (**B**) The example of current traces and I–V relationship of NCX in RVOT (*n* = 11) and RVA (*n* = 12) cardiomyocytes. Insets show the clamp protocol. *P < 0.05, ***P < 0.005 *versus* RVOT.

Next, we examined ionic currents accounting for the differences between RVOT and RVA in AP morphology and Ca^2+^ homoeostasis. RVOT and RVA cardiomyocytes had similar current densities of I_Na_ (Fig. 3A), which could explain the similar APA in RVOT and RVA cardiomyocytes. To identify the ionic currents responsible for the longer APD in RVOT cardiomyocytes, I_Na-Late_ and I_Ca-L_ (inward currents) and potassium channels (outward currents) have been compared. RVOT cardiomyocytes were characterized by a 73% larger I_Na-Late_ than RVA cardiomyocytes (Fig. 3B). In contrast, RVOT cardiomyocytes had smaller I_Ca-L_ than RVA cardiomyocytes (Fig. 4A), but half-inactivation potential was not different in RVOT and RVA cardiomyocytes (−18 ± 2 mV *versus* −17 ± 3 mV, *P* *>* 0.05). Similarly, kinetics of recovery from inactivation did not differ significantly between the two groups, albeit RVOT cardiomyocytes tended to recovery from inactivation faster than their RVA counterparts: the time constants of recovery at −80 mV were correspondingly 11 ± 1 msec. and 18 ± 3 msec. (*P* > 0.05). RVOT cardiomyocytes had a smaller NCX current than RVA cardiomyocytes (Fig. 4B).

**Fig. 5:**
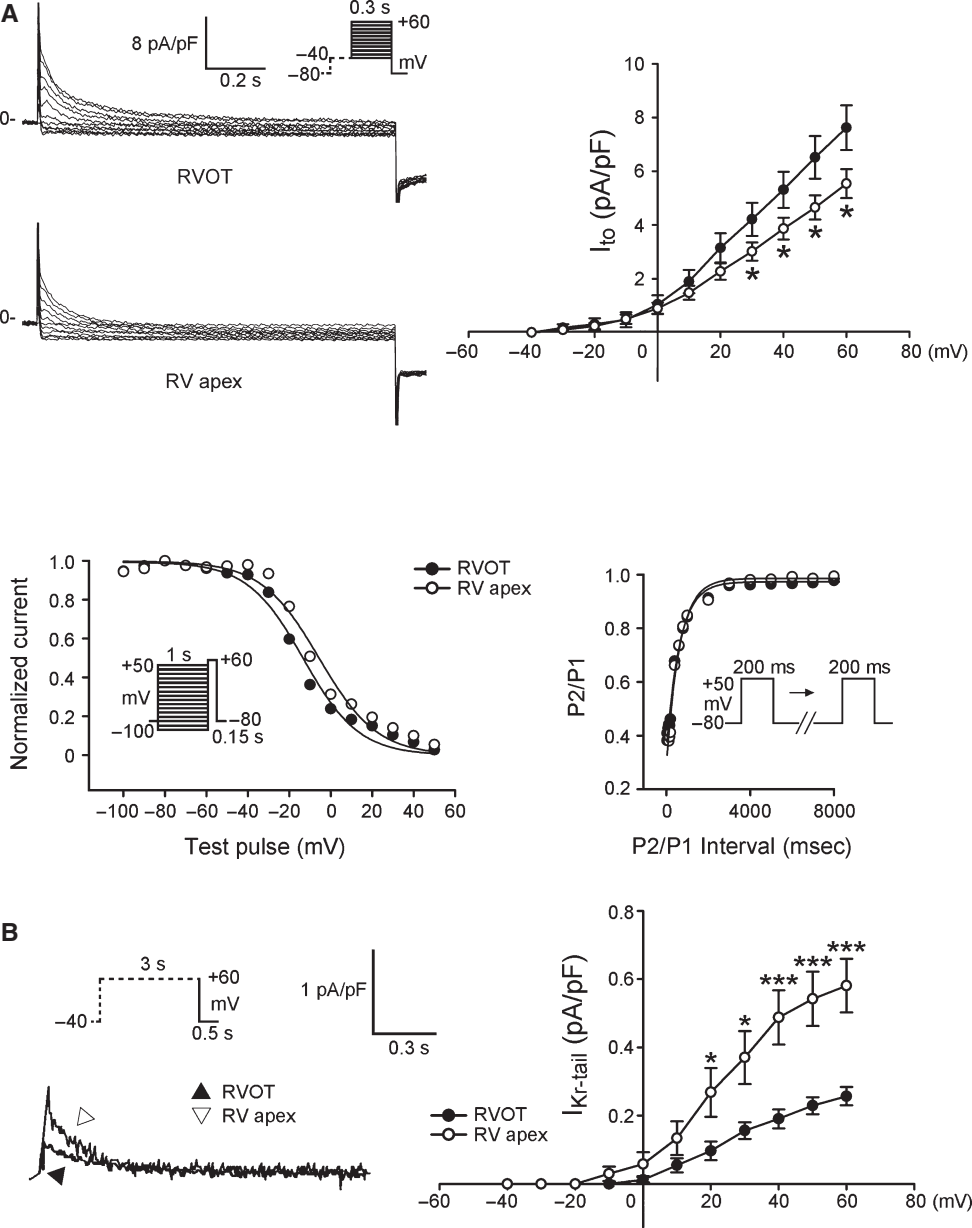
I_to_, and I_Kr-tail_ in right ventricular apex (RVA) and right ventricular out-flow tract (RVOT) cardiomyocytes. (**A**) Upper panels show the current traces and I–V relationship of I_to_ in RVOT (*n* = 16) and in RVA (*n* = 24) cardiomyocytes. Lower panels show the voltage dependence of inactivation from RVOT (*n* = 12) and RVA (*n* = 11) cardiomyocytes and the recovery kinetics of I_to_ in RVOT (*n* = 11) and in RVA (*n* = 11) cardiomyocytes. (**B**) Current traces and I–V relationship of I_Kr-tail_ in RVOT (*n* = 16) and RVA (*n* = 18) cardiomyocytes. Insets show the various clamp protocols. *P < 0.05, ***P < 0.005 *versus* RVOT.

Compared with RVA cardiomyocytes, RVOT cardiomyocytes had a larger I_to_ (Fig. 5A). RVOT cardiomyocytes were characterized by a more negative I_to_ half-inactivation potential (−14 ± 3 mV *versus* −4 ± 2 mV, *P* < 0.01) than RVA cardiomyocytes, but the time constants of recovery from inactivation for I_to_ at −80 mV were similar (742 ± 189 msec. *versus* 709 ± 186 msec., *P* > 0.05). RVOT cardiomyocytes had a smaller I_Kr-tail_ than RVA cardiomyocytes (Fig. 5B). It is feasible that the differences in the properties of ionic currents might contribute to the differences in APD and Ca^2+^ homoeostasis between RVOT and RVA cardiomyocytes.

### Arrhythmogenesis of RVOT and RVA tissue preparations

**Fig. 6:**
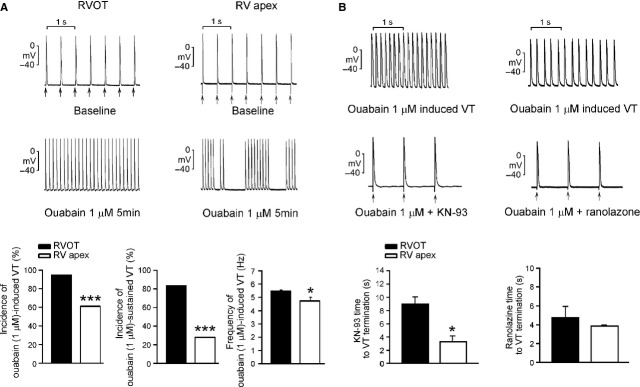
Effects of KN93 (0.1 μM) and ranolazine (0.1 μM) on ouabain-induced ventricular tachycardia (VT). (**A**) Upper and middle panels show the examples of ouabain (1 μM)-induced ventricular tachycardia (VT) in right ventricular apex (RVA) and right ventricular out-flow tract (RVOT). The lower panels show that ouabain (1 μM) induced higher incidences of total or sustained VT with a faster beating rates in the RVOT (*n* = 18) than in the RVA (*n* = 18). (**B**) Upper and middle panels show the examples that KN93 (0.1 μM) or ranolazine (0.1 μM) can terminate ouabain (1 μM)-induced sustained VT in RVOT preparations. Lower panels show that the superfusing time was longer for KN-93 (but not for ranolazine) to terminate ouabain-induced ventricular arrhythmias in the RVOT (*n* = 5) than in the RVA (*n* = 4). Arrows (↑) indicate electrical stimuli. *P < 0.05, ***P < 0.005 *versus* RVOT.

To evaluate whether there are arrhythmogenic differences between the RVOT and RVA, we studied the effects of pacing or ouabain on electrical activity in tissue preparations. Pacing-induced DADs were found in 4 of 18 RVOT preparations (22%), but not found in any of 18 RVA preparations (*P* < 0.05). These preparations were then treated with 0.1 and 1 μM ouabain. Ouabain (0.1 μM) induced a higher incidence (83.3% *versus* 50.0%, *P* < 0.005) of non-sustained (<30 sec.) VTs in the RVOT (*n* = 18) than in the RVA (*n* = 18) preparations. Moreover, ouabain (1 μM) induced more VT in the RVOT than in RVA, and the beating rates of ouabain-induced VTs were faster in the RVOT than in the RVA (Fig. 6A). In addition, ouabain (1 μM) induced a high incidence of sustained VT (>30 sec.) in the RVOT than in the RVA. Therefore, the RVOT is more susceptible to ouabain-induced ventricular arrhythmias.

### KN-93, ranolazine on ouabain-induced ventricular arrhythmias

KN-93 (0.1 μM) eliminated ouabain-induced ventricular arrhythmias in both RVOT and RVA. However, a longer KN-93 wash-in was required to terminate the ouabain-induced ventricular arrhythmias in the RVOT preparations than in the RVA preparations. Interestingly, unlike KN-93, ranolazine (0.1 μM) was able to terminate ouabain-induced VTs both in the RVOT and RVA with similar potency (Fig. 6B).

## Discussion

In this study, we found that RVOT cardiomyocytes had longer APDs than RVA cardiomyocytes, which may contribute to ventricular arrhythmias arising mainly from RVOT in patients with long QT syndrome [26,27]. I_Na-Late_ flows during the late-phase of AP to prolong APD and plays an important role in the genesis of cardiac arrhythmias as a result of enhanced triggered activity. The larger I_Na-Late_ in RVOT cardiomyocytes may alter the rate of Na^+^ extrusion/Ca^2+^ entry and lead to intracellular Ca^2+^ overload [28]. NCX plays a major role in removing intracellular Ca^2+^ and decreasing SR Ca^2+^ content. Heart failure cardiomyocytes exhibit reduced SR Ca^2+^ content and a larger NCX current, which suggests an important relationship between NCX currents and Ca^2+^ homoeostasis [29]. In our study, RVOT cardiomyocytes were characterized by a smaller NCX current, which may prevent intracellular Ca^2+^ from being driven outside the cells to maintain RVOT Ca^2+^ overload. RVOT cardiomyocytes had a faster decline in the [Ca^2+^]_i_ transient, which suggests that SR uptake is faster in RVOT cardiomyocytes. Although faster Ca^2+^ uptake into SR may potentially reduce the incidence of arrhythmia because of altered Ca^2+^ cycling, a higher SR Ca^2+^ load may also lead to spontaneous Ca^2+^ wave and produce triggered activity of DAD [30]. Ca^2+^ overload can reduce I_Ca-L_ in RVOT cardiomyocytes through Ca^2+^-dependent inactivation of I_Ca-L_, which plays a role in inhibition of Ca^2+^ influx into the cells [31]. Similar to the previous study, we found that RVOT cardiomyocytes had larger I_to_ than RVA cardiomyocytes [32]. The larger I_to_ in RVOT cardiomyocytes may mediate a more prominent phase 1 notch in AP morphology because of repolarization from K^+^ channel activation, which has been reported by Sun and Wang [33]. As I_to_ plays a pivotal role in genesis of Brugada syndrome, the larger I_to_ in RVOT cardiomyocytes may result in a high arrhythmogenesis in those patients [4]. Compared with RVA cardiomyocytes, RVOT cardiomyocytes had a more negative I_to_ half-inactivation potential, which suggests a different kinetic property of I_to_ in these cells. Altering I_to_ selectivity by using a ‘dynamic-clamp’ in previous studies showed an inverse correlation between I_to_ and I_Ca-L_ or APD [34,35]. However, this study only showed a consistent finding on I_Ca-L_, but not on APD. In contrast, other studies showed a direct correlation of I_to_ and I_Ca-L_ [36,37]. Moreover, the smaller I_Kr-tail_ in RVOT cardiomyocytes may produce a delay in phase 3 repolarization of the AP to result in their longer APD. A previous study from Nademanee *et al*. showed that the underlying electrophysiological mechanism in patients with Brugada syndrome is delayed depolarization over the anterior aspect of RVOT epicardium [38]. They found delayed depolarization in RVOT epicardium, but not in RVOT endocardium. However, this study only compared the difference between RVOT and RVA cardiomyocytes, and did not demonstrate the differences between epicardial and endocardial cardiomyocytes from the RVOT and RVA. Moreover, a relatively small sample size may limit the power of this study, and it is unclear whether our findings can be applied to pathological ventricles.

Ouabain inhibits the Na^+^-K^+^ pump, and causes Ca^2+^ overload in ventricular myocytes [39], which induces DADs and sustained VTs *in vivo* [40]. Rapid pacing induces heterogeneities in intracellular Ca^2+^ handling, which may increase [Ca^2+^]_i_ and triggered activity [41]. However, the susceptibility to triggered activity or VTs in RVOT cardiomyocytes under ouabain infusion or rapid pacing remains to be elucidated. We found that KN-93 could eliminate ouabain-induced ventricular arrhythmias in the RVOT and RVA, which suggests that CaMKII inhibition ameliorates ouabain-induced Na^+^ and Ca^2+^ overload. We found that a longer KN-93 superfusing time was required to ameliorate ouabain-induced ventricular arrhythmias in the RVOT than in the RVA. As the perfusion rate of KN-93 and tissue size of the RVOT and RVA were similar, the different time to arrhythmia termination suggests that the higher RVOT arrhythmogenesis may be caused by Ca^2+^ overload. We also found that I_Na-Late_ inhibition by ranolazine eliminated ouabain-induced VTs. This finding suggests that intracellular Ca^2+^ overload by application of ouabain can increase I_Na-Late_. The similar administration periods of ranolazine required to terminate ouabain-induced VTs in the RVOT and RVA suggest a substantial antiarrhythmic effect of ranolazine. However, ranolazine at 0.1 μM may not differentially suppress ouabain-induced ventricular arrhythmia in the RVOT and RVA, as we used a rather low concentration of ranolazine (with more specific inhibition on I_Na-Late_) to avoid its non-specific ionic effects [14].

In conclusion, our findings showed that RVOT cardiomyocytes possess distinctive electrophysiological characteristics with a longer APD and higher Ca^2+^ content. These differences may contribute to the high RVOT arrhythmogenicity.
